# Fractal dimension and cortical indices of the mandible in hypercholesterolaemia: a retrospective study

**DOI:** 10.1186/s12903-026-07817-6

**Published:** 2026-02-05

**Authors:** Dóra Iványi, Márton Kivovics, Csilla Szerencse, Orsolya Németh

**Affiliations:** Department of Public Dental Health, Semmelweis University, Szentkirályi street 40. 1088, Budapest, Hungary

**Keywords:** Fractal dimension, Lacunarity, Mandibular cortical width, Panoramic mandibular index, Panoramic radiograph, Hypercholesterolaemia, Trabecular bone microarchitecture

## Abstract

**Background:**

Hypercholesterolaemia has been associated with changes in bone metabolism, but its potential impact on mandibular bone microarchitecture, as observed in dental panoramic radiographs, remains unclear. Understanding these relationships may improve pre-implant assessment and treatment planning. This study aimed to investigate whether fractal dimension, lacunarity, mandibular cortical width, and the panoramic mandibular index differ among patients with varying serum total cholesterol levels.

**Methods:**

This retrospective study analysed panoramic radiographs from adult patients referred for dental implant treatment. Participants were stratified into three groups based on total cholesterol level: normal (< 5.2 mmol/L), borderline high (5.2–6.2 mmol/L), and high (> 6.2 mmol/L). Standardised 64 × 64 pixel regions of interest were selected in anterior, premolar, and molar mandibular regions. Fractal dimension and lacunarity were calculated using a box-counting approach, while mandibular cortical width and the panoramic mandibular index were measured manually at the mental foramen. Group comparisons were performed using the Kruskal-Wallis test with Bonferroni-corrected post-hoc analysis.

**Results:**

A total of 92 patients were included (35 normal, 33 borderline, 24 high cholesterol). Although the Kruskal-Wallis test indicated a difference in fractal dimension among cholesterol groups (*p* = 0.047), the post-hoc analysis revealed only a borderline significance between the high and normal groups (adjusted *p* = 0.050). Therefore, this finding should be interpreted cautiously. No significant differences were observed in the premolar or molar regions, or in lacunarity, mandibular cortical width, or panoramic mandibular index.

**Conclusions:**

Findings suggest that elevated cholesterol levels may be associated with subtle alterations in trabecular bone microarchitecture in the anterior mandible. Fractal analysis of panoramic radiographs could provide a non-invasive adjunct for early detection of bone changes in patients with lipid imbalance, potentially supporting individualised implant treatment planning. However, these observations should be interpreted with caution, and further studies are needed to confirm the reproducibility and clinical relevance of these findings.

**Trial registration:**

Not applicable.

**Supplementary Information:**

The online version contains supplementary material available at 10.1186/s12903-026-07817-6.

## Background

In contemporary dental implantology, assessing bone quality is essential for achieving predictable osseointegration and long-term implant success [1]. While conventional radiographic evaluations provide two-dimensional structural information, they often fail to capture the complexity of trabecular bone architecture, which plays a key role in primary stability and bone remodelling [2].

There are several methods for assessing bone quality, one of which is fractal analysis (FA), a reliable and non-invasive approach [3]. FA is a mathematical method used to evaluate complex and irregular geometric structures, such as trabecular bone [3]. Its primary quantitative parameter, the fractal dimension (FD), describes how completely a structure fills space and reflects its degree of complexity and self-similarity [3]. Mathematically, FD can be expressed as D = log n / log ε, where *n* is the number of self-repeating elements and *ε* is the scale of reduction [4, 5]. Digital radiographic images of trabecular bone are suitable for FA, provided that appropriate preprocessing is applied [6].

Clinical studies have linked FD to primary implant stability, including implant stability quotient (ISQ) value [7, 8]. Moreover, decreased FD values have been reported in systemic conditions such as osteoporosis [9–11] and osteoarthritis [12], highlighting its potential role in pre-implant bone quality assessment.

Lacunarity complements FD by describing the distribution and size of gaps within trabecular bone, offering further insight into bone microarchitecture [13, 14]. Although it is less studied than FD in dentistry, lacunarity has demonstrated diagnostic potential in detecting porous or disrupted bone patterns that may otherwise go unnoticed in standard fractal evaluations [13]. Because trabecular bone exhibits fractal-like behavior, FD and lacunarity can serve as indicators of potential metabolic alterations affecting bone quality and skeletal health.

Mandibular cortical parameters also contribute to radiographic assessment of bone status. Mandibular cortical width (MCW), typically measured at the mental foramen, has been correlated with systemic bone loss, especially in postmenopausal or osteoporotic women [10, 15]. The panoramic mandibular index (PMI), which standardises MCW against mandibular height, helps to mitigate the effects of image magnification and is considered a stable indicator of bone quality of the skeleton [15, 16].

Despite the relevance of these imaging markers, little is known about how metabolic conditions – particularly lipid imbalance – might influence them. Studies suggest that hypercholesterolaemia alters bone remodelling by promoting osteoclastic activity, impairing osteoblast function, and increasing oxidative stress [17]. These changes may have implications for implant outcomes, as elevated cholesterol has been linked to delayed osseointegration and peri-implant complications [18, 19]. Exploring how lipid profiles relate to mandibular bone architecture may offer a non-invasive route for identifying patients at higher skeletal risk prior to implant placement.

The aim of this study was to determine whether FD, lacunarity, MCW, and PMI vary among patients with differing serum cholesterol levels. By integrating radiomorphometric and biochemical data, this research seeks to clarify whether systemic lipid status is reflected in mandibular bone structure, with potential relevance for clinical decision-making in implant planning.

The null hypothesis of this study is that there is no statistically significant difference in the values of FD, lacunarity, MCW, or PMI between patients with normal and elevated cholesterol levels prior to dental implant placement.

## Methods

### Participants

This retrospective case-control study was approved by the Regional Research Ethics Committee of Semmelweis University (approval number: 189/2024) and conducted in accordance with the Declaration of Helsinki. Reporting adhered to the Strengthening the Reporting of Observational studies in Epidemiology (STROBE) guidelines, with the checklist provided in Additional file 1. Participants were recruited from patients treated at Department of Public Dental Health, Semmelweis University, between 2015 and 2025. The collection and analysis of data was conducted in January 2025. Written informed consent was obtained from all patients.

*Inclusion criteria were as follows:*

- Aged 18 years or older.
- Indication for dental implant therapy.
- Availability of preoperative laboratory tests including serum cholesterol.

*Exclusion criteria included:*

- Uncontrolled systemic disorders known to affect bone metabolism, such as osteoporosis, Cushing’s syndrome, hypophosphatasia, osteogenesis imperfecta, osteomalacia, Paget’s disease, osteopenia, osteofibrosis, hyperparathyroidism, hypophosphatemia, vitamin D deficiency, skeletal dysplasias, chronic kidney disease, or poorly managed diabetes mellitus.
- Current or past use of medications influencing bone turnover, including antiresorptive agents (e.g., bisphosphonates, denosumab, monoclonal antibodies, VEGF inhibitors), systemic glucocorticoids, calcitonin, parathyroid hormone analogues, long-term heparin therapy, cyclosporine, high-dose medroxyprogesterone acetate, chemotherapeutic drugs (e.g., methotrexate, ifosfamide, imatinib), thiazolidinediones, or antiretroviral treatments.
- History of chemotherapy.
- History of therapeutic irradiation involving the head or neck regions.
- Presence of untreated or persistent periapical pathology.
- Signs of active or uncontrolled periodontal disease.
- Use of recreational drugs.
- Ongoing anticoagulant therapy that would contraindicate surgical procedures.
- Documented psychological disorders, physical or cognitive impairment.
- Excessive alcohol consumption or heavy tobacco use.
- Pregnancy or lactation.

Patients were categorized into three groups based on their total cholesterol levels, measured in mmol/L, following NCEP ATP III criteria [20] and Mayo Clinic’s guideline [21]:

- Normal: <5.2 mmol/L.
- Borderline high: 5.2–6.2 mmol/L.
- High: >6.2 mmol/L.

Before implant placement, all patients underwent full oral sanitation, including extraction of hopeless teeth, periodontal treatment, and necessary restorations. Panoramic radiographs and cone-beam CT scans were also obtained for treatment planning.

### Laboratory assessment

All laboratory investigations were performed in accredited medical laboratories through the national primary care (general practitioner) system. These tests were not part of routine population screening but were requested to evaluate systemic factors potentially affecting the success and prognosis of dental implant therapy.

The laboratory panel included:

- Complete blood count (including white and red blood cell quantity and morphology).
- Inflammatory markers: C-reactive protein (CRP), erythrocyte sedimentation rate (ESR).
- Glucose metabolism: fasting blood glucose, haemoglobin A1c (HbA1c).
- Lipid profile: total cholesterol, triglycerides, low-density lipoprotein (LDL), high-density lipoprotein (HDL).
- Liver function tests: alanine aminotransferase (ALT), aspartate aminotransferase (AST), alkaline phosphatase (ALP), gamma-glutamyl transferase (GGT), bilirubin.
- Renal function panel: blood urea nitrogen (BUN), creatinine, estimated glomerular filtration rate (eGFR), electrolytes (Na⁺, K⁺, Cl⁻, Ca²⁺).
- Bone metabolism markers: ALP (bone-specific fraction), osteocalcin, parathyroid hormone (PTH), serum C-terminal telopeptide (CTx).
- Iron status: serum iron, transferrin saturation, total iron binding capacity (TIBC).
- Complete metabolic panel: albumin, total protein, creatine phosphokinase (CPK).

Not all patients had data available for every parameter. Some patients were also tested for additional markers such as thyroid-stimulating hormone (TSH), rheumatoid factor, or 25-hydroxy vitamin D, based on medical indication.

These laboratory parameters were chosen for their established or suspected association with bone remodelling, metabolic bone disease, or peri-implant complications.

### Radiographic image analysis

Panoramic radiographs were acquired using a KAVO OP 3D Pro unit (KAVO, Biberach an der Riß, Germany) under standardized exposure settings: 66.42 kV, 10 mA, 16.18 s exposure time, 50 cm focus-to-detector distance. Prior to conducting fractal analysis and lacunarity measurements, all orthopantomographic images underwent standardized preprocessing steps to ensure uniformity and suitability for further evaluation. Image processing and analysis were carried out using ImageJ software (version 1.54p, bundled with 64-bit Java 1.8.0_322; developed by W. Rasband, National Institutes of Health, Bethesda, MD, USA; available at https://imagej.nih.gov/ij) [22].

Measurements were performed on regions of interest (ROIs) on the right side of the mandible. In instances where inflammation precluded the use of the right side, the ROIs on left side were designated for image analysis. For each patient, three ROIs were measured: the front, premolar and molar regions. The ROIs had a size of 64 × 64 pixels, and only trabecular bone was included within these ROIs. In the anterior region, the regions of interest (ROIs) were placed apically from the roots of the lateral incisors of the right mandible, in the trabecular bone, excluding cortical and pathological structures. In the premolar region, the regions of interest were placed apically from the roots of the premolars, taking care to exclude the mental foramen. In the molar region, the ROIs were located below the roots of the second molars. In edentulous mandibular regions, anatomical orientation was based on the mental foramen as a reference landmark for region identification, while ensuring that the foramen itself was not included within the selected ROI. Due to their confounding effect on FA tooth, implant, anatomical features, inflammation, or other bone lesions could not be included in the ROIs. Image analysis was performed in accordance with the method established by Rudolph and White [6]. Following ROI cropping, the images were saved in TIFF format and duplicated. The X-ray brightness due to soft tissue and bone thickness was reduced on the duplicated ROI using a Gaussian blur (sigma = 35). The Gaussian filtered version was then subtracted from the original cropped image, after which 128 grey values were added to each pixel. The image was binarized by establishing a threshold of 128 in order to effectively differentiate between the trabecular bone and lacunae. Therefore, pixels in the displayed image with a grey value lower than 128 were black, while pixels with a grey value higher than 128 were white. The image was then eroded and dilated to ensure that reduce radiological noise. Finally, the image was inverted and skeletonized, resulting in a pattern that matches the trabecular bone structure.

**Fig. 1:**
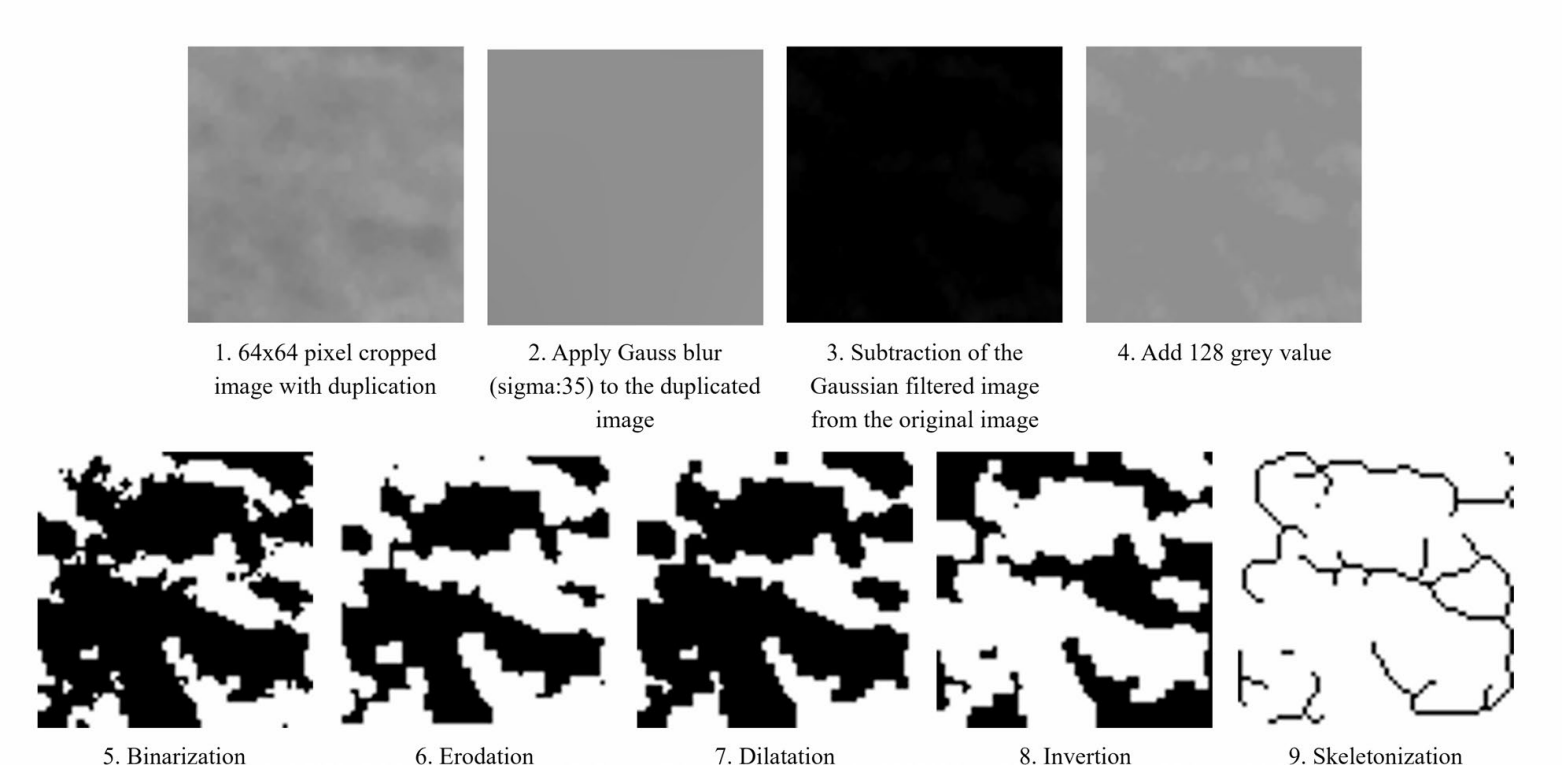
Image preprocessing steps for panoramic radiographs. Illustrative sequence of preprocessing applied before fractal analysis. A 64 × 64 pixel region of interest was selected, Gaussian blur applied, and the blurred image subtracted from the original. Contrast was normalized, followed by binarization, erosion, dilatation, inversion, and skeletonization. Processing was performed using ImageJ with the FracLac plugin

The fractal analysis and the lacunarity were calculated using the FracLac plugin (version 2.5, A. Karperien) for ImageJ [23]. The fractal analysis was performed using the box-counting method (pixel sizes: 2, 3, 4, 6, 8, 12, 16, 32, 64), in which the number of boxes required to cover the binarized trabecular pattern is counted across multiple scales. The slope of the regression line between log(box size) and log(number of boxes) represents the FD value, while lacunarity was computed based on the variation in pixel occupancy within these boxes, reflecting trabecular heterogeneity [6]. (Fig. 1.)

In addition to fractal dimension and lacunarity, the following radiomorphometric parameters were evaluated using panoramic radiographs:

#### Mandibular cortical width (MCW)

**Fig. 2:**
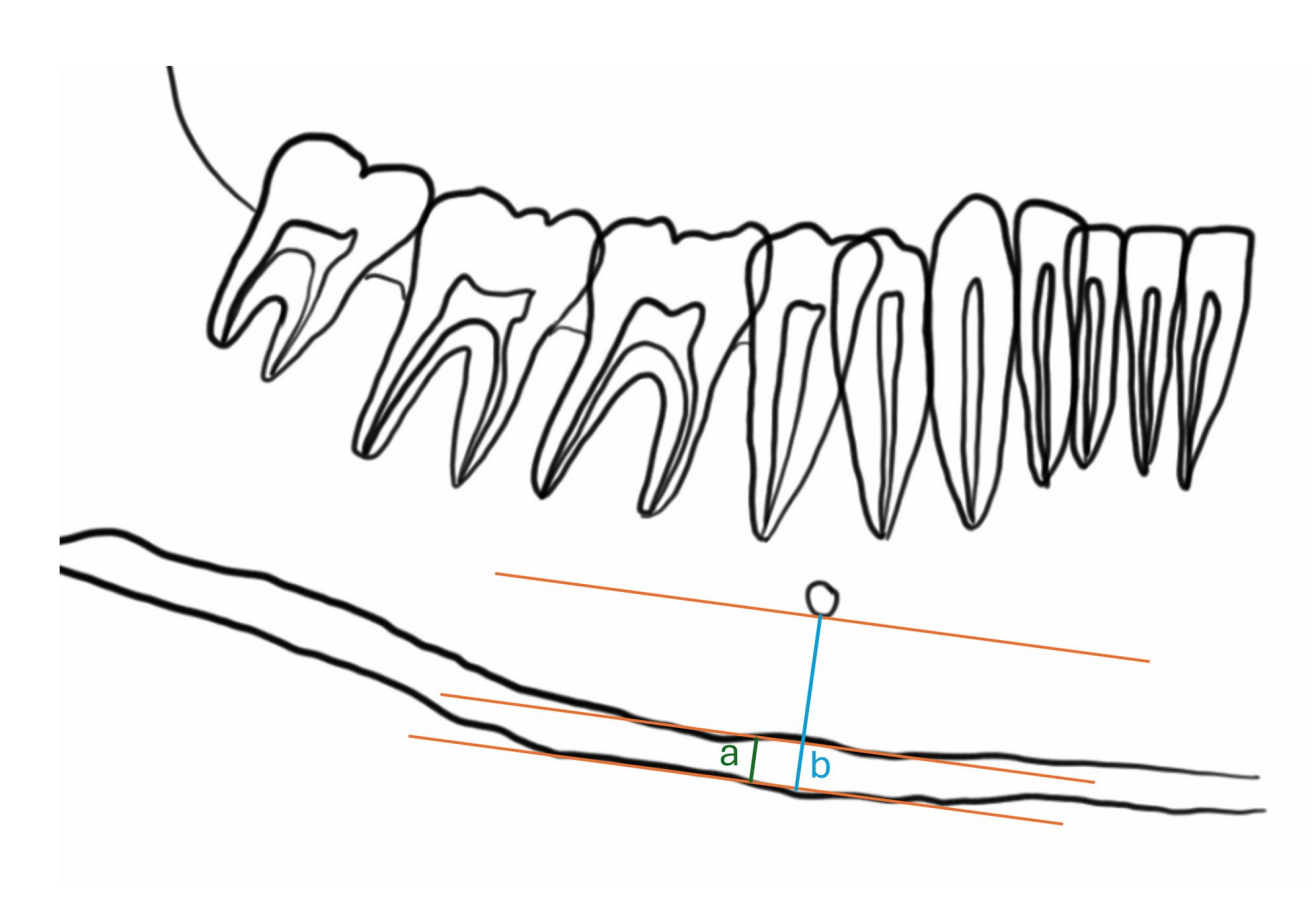
The image shows the measurements needed to determine the Mandibular Cortical Width, MCW (**a**) and the Panoramic Mandibular Index (PMI) (**a**/**b**). Digital panoramic radiographs showing the mental foramen region. Mandibular cortical width (MCW) is measured as the cortical thickness perpendicular to the mandible’s lower border through the mental foramen. The panoramic mandibular index (PMI) is calculated as MCW divided by the distance from the lower edge of the mental foramen to the inferior mandibular border

The MCW was measured at the mental foramen region as the cortical bone thickness on a line perpendicular to the lower border of the mandible passing through the lower edge of the mental foramen. This measurement serves as an indicator of mandibular bone quality and is widely used in the assessment of osteoporosis and systemic bone loss [15]. (Fig. 2.)

#### Panoramic mandibular index (PMI)

The PMI was calculated as the ratio between the mandibular cortical width at the mental foramen region and the distance from the lower edge of the mental foramen to the inferior border of the mandible. This index normalizes cortical width for mandibular height and has been shown to correlate with skeletal bone status [15]. (Fig. 2.)

All measurements were performed using ImageJ software. The radiographs were acquired under standardized exposure settings and reviewed for diagnostic quality prior to analysis. Pixel-based values were used, as validated in prior morphometric studies.

To ensure measurement consistency, a single trained examiner conducted all analyses under standardized display and lighting conditions using identical software settings. Although formal intra- and inter-observer reliability testing was not conducted, consistent methodological parameters were applied across all cases to ensure reproducibility.

### Statistical analysis

Statistical analysis was performed using IBM SPSS Statistics software, version 30.0 (IBM Corporation, New York, NY, USA). Normality was assessed via Kolmogorov-Smirnov and Shapiro-Wilk tests. As the majority of the variables did not follow a normal distribution, non-parametric statistical methods were applied throughout the analysis.

Differences among the cholesterol-level groups were analysed using the Kruskal-Wallis test, followed by Dunn’s post hoc test with Bonferroni correction to identify pairwise differences. All statistical results were considered statistically significant at *p* < 0.05.

In addition, multiple linear regression analyses were performed to assess the potential confounding effects of age and sex on fractal dimension (FD) values in the anterior, premolar, and molar regions.

All individual-level and summary data used in the analysis are openly available at Mendeley Data: Iványi, Dóra; Kivovics, Márton; Szerencse, Csilla; Németh, Orsolya (2025), “Radiographic and Laboratory Dataset for Fractal Dimension and Cortical Indices of the Mandible in Hypercholesterolaemia”, Mendeley Data, V1, doi: 10.17632/48fkkf4v2r.1.

## Results

**Fig. 3:**
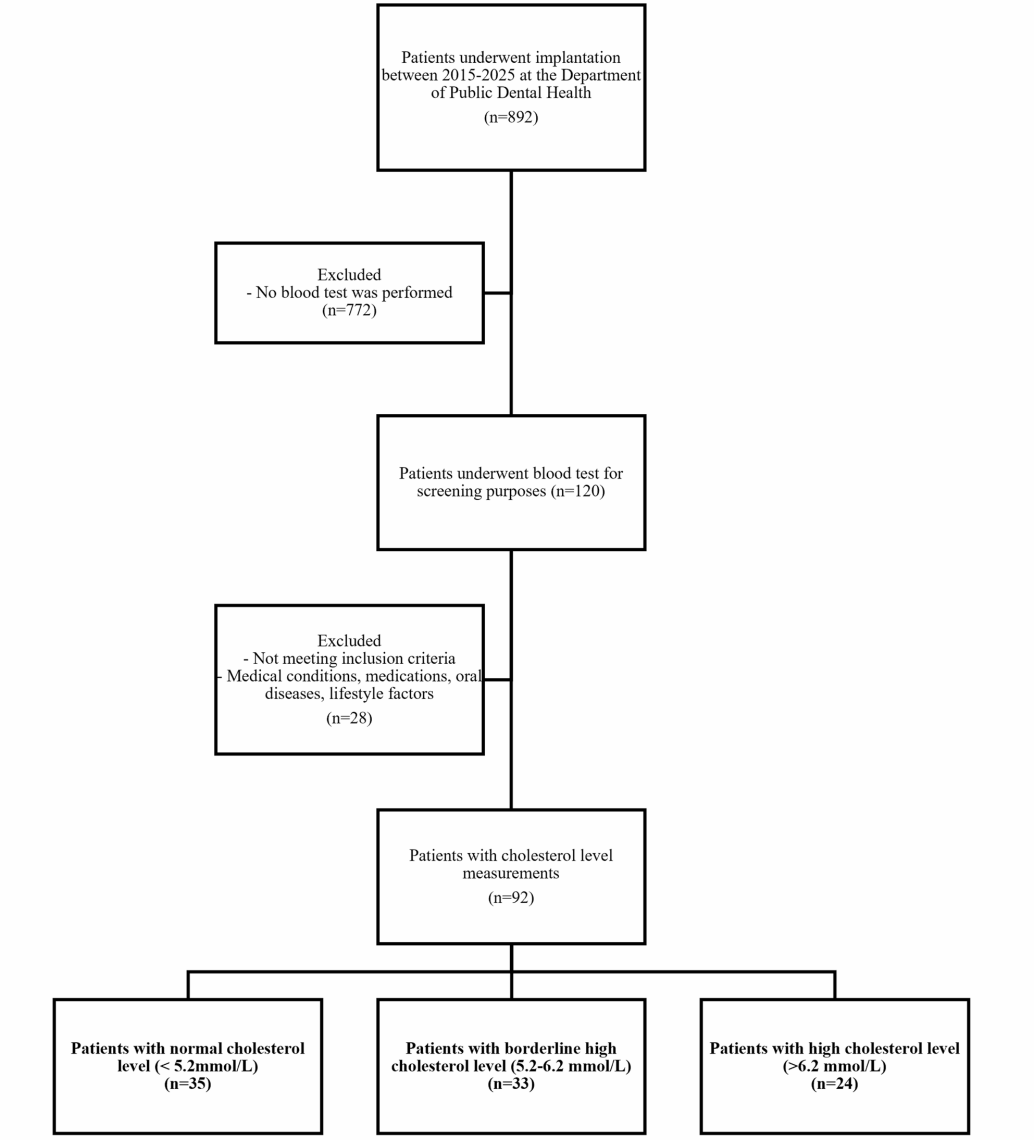
Patient recruitment and selection flowchart. Flowchart showing patient inclusion and exclusion process. Criteria applied to initial pool resulted in the final sample of 92 patients, stratified into normal, borderline high, and high cholesterol groups

The patient selection criteria are shown in the flowchart below. (Fig. 3.)

A total of 92 patients were included in the study and categorized into three groups based on total serum cholesterol levels: normal (< 5.2 mmol/L, *n* = 35), borderline high (5.2–6.2 mmol/L, *n* = 33), and high (> 6.2 mmol/L, *n* = 24). The mean ages were 60.4 ± 19.0, 62.7 ± 14.4, and 68.88 ± 8.2 years, respectively. Table 1. shows the descriptive data for the examined groups.

**Table 1:** Descriptive data for the population

Variable	Normal (< 5.2 mmol/L)	Borderline (5.2–6.2 mmol/L)	High (> 6.2 mmol/L)
N	35	33	24
Male	16 (45.7%)	15 (45.5%)	3 (12.5%)
Female	19 (54.3%)	18 (54.5%)	21 (87.5%)
Age	60.4 ± 19.0	62.7 ± 14.4	68.88 ± 8.2

Values are presented as mean ± standard deviation for continuous variables and number (percentage) for categorical variables. Groups were defined according to total serum cholesterol: Normal (<5.2 mmol/L), Borderline high (5.2–6.2 mmol/L), High (>6.2 mmol/L)

Frontal FD values showed a statistically significant difference between the groups (Kruskal-Wallis, *p* = 0.047). The median FD was 1.4192 [1.0977–1.5935] in the normal group, 1.4273 [1.1339–1.6019] in the borderline group, and 1.4771 [1.2576–1.5785] in the high cholesterol group. Post hoc comparisons (Dunn-Bonferroni) indicated a borderline significant difference between the normal and high cholesterol groups (adjusted *p* = 0.050), while no significant differences were found between the normal and borderline, or borderline and high groups.

In the premolar region, median FD values were 1.5100 [1.2381–1.5789], 1.5060 [1.2254–1.8820], and 1.5045 [1.3373–1.6171] in the normal, borderline, and high cholesterol groups, respectively (*p* = 0.988). In the molar region, medians were 1.5180 [1.2549–1.5878], 1.5062 [1.2427-1.6100], and 1.5305 [1.2928–1.6330], respectively (*p* = 0.378). These differences were not statistically significant.

Lacunarity values showed no significant differences among groups in any region. In the frontal region, medians were 0.2036 [0.1130–0.3319], 0.1906 [0.1235–0.3145], and 0.1866 [0.1116-0.3200] for the normal, borderline, and high groups, respectively (*p* = 0.424). In the premolar region, medians were 0.1805 [0.1240–0.2962], 0.1630 [0.1347–0.3246], and 0.1702 [0.0947–0.2472] (*p* = 0.336). In the molar region, values were 0.1774 [0.1114–0.3288], 0.1815 [0.1264–0.4014], and 0.1650 [0.1142–0.2694] (*p* = 0.300).

No statistically significant differences were found in MCW values among the groups on either side of the mandible. On the right side, the median MCW was 47.50 [27.90-65.92] in the normal group, 47.70 [33.20–62.30] in the borderline group, and 45.25 [23.40–86.60] in the high cholesterol group (*p* = 0.413). On the left side, values were 49.00 [30.70–71.20], 46.70 [32.60-64.34], and 43.65 [22.20-89.64], respectively (*p* = 0.430).

PMI values also did not differ significantly between groups. On the right side, medians were 0.3670 [0.2255–0.5677] in the normal group, 0.3609 [0.2312–0.7467] in the borderline group, and 0.3687 [0.1753–0.7205] in the high group (*p* = 0.556). On the left side, median PMI values were 0.3585 [0.1930–0.5249], 0.3497 [0.1955–0.6343], and 0.3568 [0.1832–0.7207], respectively (*p* = 0.948).

All statistical comparisons are summarized in Tables 2 and 3.

**Table 2:** Fractal dimension and lacunarity in different mandibular regions across cholesterol-level groups

Region	Measure	Normal (< 5.2 mmol/L)	Borderline high (5.2–6.2 mmol/L)	High (> 6.2 mmol/L)	*p*-value
Front	Median FD	1.4192	1.4273	1.4771	**0.047** ^1*^
	Range Width	0.4958	0.4680	0.3209	
	Minimum	1.0977	1.1339	1.2576	
	Maximum	1.5935	1.6019	1.5785	
	Median Lacunarity	0.2036	0.1906	0.1866	0.424^1^
	Range Width	0.2189	0.1910	0.2084	
	Minimum	0.1130	0.1235	0.1116	
	Maximum	0.3319	0.3145	0.3200	
Premolar	Median FD	1.5100	1.5060	1.5045	0.988^1^
	Range Width	0.3408	0.6566	0.2798	
	Minimum	1.2381	1.2254	1.3373	
	Maximum	1.5789	1.8820	1.6171	
	Median Lacunarity	0.1805	0.1630	0.1702	0.336^1^
	Range Width	0.1722	0.1899	0.1525	
	Minimum	0.1240	0.1347	0.0947	
	Maximum	0.2962	0.3246	0.2472	
Molar	Median FD	1.5180	1.5062	1.5305	0.378^1^
	Range Width	0.3329	0.3673	0.3402	
	Minimum	1.2549	1.2427	1.2928	
	Maximum	1.5878	1.6100	1.6330	
	Median Lacunarity	0.1774	0.1815	0.1650	0.300^1^
	Range Width	0.2174	0.2750	0.1552	
	Minimum	0.1114	0.1264	0.1142	
	Maximum	0.3288	0.4014	0.2694	

^1^ Kruskal-Wallis test ^*^Significant p-value

Values are median [minimum-maximum]; FD, fractal dimension; lacunarity, measure of trabecular bone heterogeneity; p-values calculated using Kruskal-Wallis test; pairwise comparisons performed with Dunn’s post-hoc test and Bonferroni correction. *p < 0.05

**Table 3:** Mandibular cortical width (MCW) and panoramic mandibular index (PMI) values by cholesterol-level group

Variable	Side	Variable	Normal (< 5.2 mmol/L)	Borderline high (5.2–6.2 mmol/L)	High (> 6.2 mmol/L)	*p*-value
MCW	Right	Median	47.50	47.70	45.25	0.413^1^
		Range Width	38.02	29.10	63.20	
		Minimum	27.90	33.20	23.40	
		Maximum	65.92	62.30	86.60	
	Left	Median	49.00	46.70	43.65	0.430^1^
		Range Width	40.50	31.74	67.44	
		Minimum	30.70	32.60	22.20	
		Maximum	71.20	64.34	89.64	
PMI	Right	Median	0.3670	0.3609	0.3687	0.556^1^
		Range Width	0.3420	0.5160	0.5450	
		Minimum	0.2255	0.2312	0.1753	
		Maximum	0.5677	0.7467	0.7205	
	Left	Median	0.3585	0.3597	0.3568	0.948^1^
		Range Width	0.3320	0.4390	0.5380	
		Minimum	0.1930	0.1955	0.1832	
		Maximum	0.5249	0.6343	0.7207	

^1^ Kruskal-Wallis test

Values are median [minimum-maximum]; MCW, mandibular cortical width; PMI, panoramic mandibular index; p-values calculated using Kruskal-Wallis test; pairwise comparisons performed with Dunn’s post-hoc test and Bonferroni correction

To further evaluate the influence of demographic variables, multiple regression analyses including age and sex as covariates were conducted for each mandibular region.

Neither age nor sex showed a statistically significant association with FD values (anterior: age *p* = 0.067, sex *p* = 0.661; premolar: age *p* = 0.657, sex *p* = 0.531; molar: age *p* = 0.062, sex *p* = 0.135).

## Discussion

This retrospective study investigated whether mandibular radiographic indices – fractal dimension (FD), lacunarity, mandibular cortical width (MCW), and panoramic mandibular index (PMI) – change according to serum total cholesterol levels. Among these parameters, only FD in the anterior mandibular region showed a statistically significant increase in the high-cholesterol group compared to the normal group. Although the Kruskal-Wallis test indicated a significant difference in anterior FD across cholesterol groups (*p* = 0.047), the post hoc result was borderline (adjusted *p* = 0.050), suggesting a weak group-level effect. No significant differences were found in MCW, PMI, or lacunarity.

Since FD reflects trabecular complexity and connectivity, higher values may indicate either compensatory remodelling or structural disorganization rather than improved bone quality. The anterior mandible, characterized by thin cortical plates and a dense trabecular matrix, may be more responsive to systemic metabolic influences than the posterior mandible, which is more cortical-dominant [24–26]. Our standardized and region-specific methodology – using consistent ROIs and preprocessing protocols – likely minimized anatomical and image-related biases. This supports the interpretation that the increased FD observed in hypercholesterolaemic patients reflects a real skeletal alteration, not an imaging artifact.

While radiomorphometric changes in mandibular bone have been extensively studied in conditions like osteoporosis [6, 9, 27] and osteoarthritis [12], the effect of lipid metabolism remains less explored. Günacar et al. [28] and Serindere et al. [29] both reported significant alterations in FD and cortical measurements in hyperlipidaemic patients, especially in the premolar and molar regions. In contrast, our study detected increased FD only in the anterior region, without significant differences in other areas or in cortical indices. Several factors may explain these discrepancies. Differences in anatomical focus and image analysis techniques likely contributed. Additionally, numerous unmeasured biological and clinical variables could have influenced bone architecture: menopausal status [30], the duration of edentulism [13, 31], and underlying hormonal milieu [32–36] all affect skeletal remodelling. Our study did not stratify by these factors. The time course and management of hypercholesterolaemia also varied among participants, some patients may have had untreated, long-standing dyslipidaemia, while others were under regular medical care or receiving lipid-lowering therapy with varying adherence. The accuracy of medical histories and differences in systemic disease control introduce further heterogeneity.

Given the metabolic activity and trabecular density of the anterior mandible [24, 25], our ROI placement may have been especially sensitive to detecting early changes in bone structure. Unlike osteoporosis, where FD typically decreases due to trabecular thinning [6, 27], hyperlipidaemia may cause more complex remodelling patterns, potentially resulting in increased FD. At the cellular level, lipid accumulation and oxidative stress can stimulate osteoclastic bone resorption while inhibiting osteoblast differentiation, leading to an imbalance between bone formation and resorption [77,84]. These mechanisms are further amplified by increased production of pro-inflammatory cytokines and endothelial dysfunction, both of which impair local microcirculation and bone regeneration [37–39]. Such metabolic disturbances may contribute to altered trabecular organization and higher FD values, even in the absence of overt bone loss. However, without histological or longitudinal validation, the biological meaning of these findings remains speculative. These results reinforce the importance of interpreting FD within a condition-specific framework and emphasize that skeletal responses to systemic conditions may vary by region. Despite the limited literature in this area, our findings suggest that subtle trabecular alterations related to lipid imbalance may be captured using radiographic complexity metrics.

FD analysis on panoramic radiographs may serve as a non-invasive adjunct to detect early trabecular changes in patients with dyslipidaemia. Given the importance of bone quality in implant success [27, 40, 41], identifying patients with subtle skeletal vulnerabilities could support more personalized risk assessment and treatment planning. The anterior mandible, due to its accessibility and trabecular composition, may be a practical region of interest. However, further studies are needed to clarify whether this region uniquely reflects systemic lipid-related changes or simply offers improved radiographic resolution for trabecular metrics.

Still, elevated FD should not be assumed to indicate superior bone quality. In systemic disease contexts, it may reflect irregular or dysregulated remodelling. Therefore, FD should be interpreted as a supportive metric rather than a stand-alone diagnostic tool.

Furthermore, to account for demographic variability, we controlled for sex and age using multiple linear regression. These analyses confirmed that neither variable significantly affected FD values, indicating that the observed differences between cholesterol groups were not confounded by age or sex distribution.

Taken together, these findings support the hypothesis that systemic lipid alterations may influence bone metabolism, although the direction and magnitude of this effect appear to vary across studies and skeletal sites. Our results, showing increased FD in hypercholesterolaemic patients, may indicate a distinct phase or regional response of trabecular bone remodelling to lipid imbalance.

This study has several limitations. Its retrospective design limits causal inference. The sample size, though adequate for primary analyses, may not support subgroup comparisons. As a sensitivity assessment, a post-hoc power analysis was performed for anterior fractal dimension using an ANOVA-based approximation. Based on the Kruskal-Wallis test statistic (H = 6.104; *N* = 92), the corresponding effect size was η² ≈ 0.067 (Cohen’s f ≈ 0.27), yielding an achieved power of 0.62, indicating moderate sensitivity to detect small-to-moderate group differences.

Only total cholesterol was analysed; LDL, HDL, and triglycerides, which may have differential effects on bone, were not included. Cholesterol levels were assessed at a single time point, without insight into chronic exposure. In addition, medication use (e.g., statins) and systemic conditions such as diabetes were not controlled for and may have influenced bone metabolism. Moreover, we did not stratify participants by sex, menopausal status, or hormonal parameters, which are known to affect bone metabolism. This would help to clarify whether FD indeed reflects metabolically driven skeletal changes and how these findings might be reflected in implant outcomes. Manual delineation of regions of interest may represent a source of measurement-related variability; however, this approach is consistent with the methodology applied in the majority of previously published studies on fractal analysis of mandibular bone. In addition, the use of two-dimensional panoramic radiographs represents a limitation, as 2D imaging cannot fully capture three-dimensional trabecular architecture; future studies using CBCT or other 3D modalities are therefore warranted.

## Conclusions

In conclusion, our findings suggest that systemic lipid imbalance may influence trabecular bone architecture in a regionally specific manner, as reflected by increased fractal dimension in the anterior mandible. While no differences were observed in cortical indices or lacunarity, FD may serve as a sensitive, non-invasive indicator of early trabecular changes in patients with hypercholesterolaemia.

These results highlight the potential of radiographic complexity analysis in identifying skeletal lesions relevant for implant planning. Further prospective studies are needed to validate these results and to explore the utility of FD in systemic risk assessment and personalised implant therapy.

## Supplementary Information

Supplementary Material 1.

## Data Availability

The datasets generated and analysed during the current study are publicly available in the Mendeley Data repository: Iványi, Dóra; Kivovics, Márton; Szerencse, Csilla; Németh, Orsolya (2025), “Radiographic and Laboratory Dataset for Fractal Dimension and Cortical Indices of the Mandible in Hypercholesterolaemia”, Mendeley Data, V1, doi: 10.17632/48fkkf4v2r.1.

## Abbreviations

FA: fractal analysis
FD: fractal dimension
ISQ: implant stability quotient
MCW: mandibular cortical width
PMI: panoramic mandibular index
STROBE: Strengthening the Reporting of Observational studies in Epidemiology
ROI: region of interest
LDL: low-density lipoprotein
HDL: high-density lipoprotein
