# Supplementary material for: Fractal dimension and cortical indices of the mandible in hypercholesterolaemia: a retrospective study
Source: BMC Oral Health. 2026 Feb 5;26:455. doi: 10.1186/s12903-026-07817-6 (PMC12973740; doi:10.1186/s12903-026-07817-6)
Supplement: Supplementary file 1 — Supplementary Material 1. [file 12903_2026_7817_MOESM1_ESM.docx]

STROBE Statement—checklist of items that should be included in reports of observational studies

|  | Item No. | Recommendation | Page  No. | Relevant text from manuscript |
| --- | --- | --- | --- | --- |
| **Title and abstract** | 1 | (*a*) Indicate the study’s design with a commonly used term in the title or the abstract | Title | Fractal Dimension and Cortical Indices of the Mandible in Hypercholesterolaemia: A Retrospective Study |
|  |  | (*b*) Provide in the abstract an informative and balanced summary of what was done and what was found | Abstract | The structured summary concisely and accurately summarizes background information, objectives, methods (including population and analysis), results, and conclusions. |
| Introduction | | | |  |
| Background/rationale | 2 | Explain the scientific background and rationale for the investigation being reported | Introduction | The introduction describes the importance of bone quality in the success of implants and reviews the evidence suggesting that systemic lipid levels may influence bone metabolism. The rationale for examining radiological bone indices in this context is explained. |
| Objectives | 3 | State specific objectives, including any prespecified hypotheses | The final paragraph of the introduction | The null hypothesis of this study is that there is no statistically significant difference in the values of FD, lacunarity, MCW, or PMI between patients with normal and elevated cholesterol levels prior to dental implant placement. |
| Methods | | | |  |
| Study design | 4 | Present key elements of study design early in the paper | “Participants” subsection in Materials and Methods | Retrospective case–control study |
| Setting | 5 | Describe the setting, locations, and relevant dates, including periods of recruitment, exposure, follow-up, and data collection | “Participants” subsection in Materials and Methods | The study was conducted at Semmelweis University’s Department of Public Dental Health. Patients were recruited from 2015 to 2025. Data analysis was conducted in January 2025. |
| Participants | 6 | (*a*) *Cohort study*—Give the eligibility criteria, and the sources and methods of selection of participants. Describe methods of follow-up  *Case-control study*—Give the eligibility criteria, and the sources and methods of case ascertainment and control selection. Give the rationale for the choice of cases and controls  *Cross-sectional study*—Give the eligibility criteria, and the sources and methods of selection of participants | “Participants” subsection in Materials and Methods | Inclusion criteria included age over 18, indication for implant therapy, and availability of preoperative laboratory data. Exclusion criteria covered a wide range of systemic bone-affecting conditions and treatments, as well as factors that might interfere with radiographic evaluation or implant success. |
|  |  | (*b*) *Cohort study*—For matched studies, give matching criteria and number of exposed and unexposed  *Case-control study*—For matched studies, give matching criteria and the number of controls per case |  | Not applicable |
| Variables | 7 | Clearly define all outcomes, exposures, predictors, potential confounders, and effect modifiers. Give diagnostic criteria, if applicable | Methods | The outcome variables were the radiomorphometric measurements (fractal dimension, lacunarity, mandibular cortical width, and panoramic mandibular index), which were compared across cholesterol categories. Since this was a retrospective, exploratory study, no predefined clinical outcomes or endpoints were used. Exposure was defined as total serum cholesterol level, categorized according to NCEP ATP III criteria. Potential confounding factors (e.g., hormonal status, disease duration) were not controlled for but are discussed as limitations. |
| Data sources/ measurement | 8* | For each variable of interest, give sources of data and details of methods of assessment (measurement). Describe comparability of assessment methods if there is more than one group | Methods | Laboratory data were obtained from accredited national GP-based labs. Imaging data were collected via standardised panoramic radiographs, processed and analysed using ImageJ and FracLac according to validated protocols. |
| Bias | 9 | Describe any efforts to address potential sources of bias | Methods | Potential sources of bias are discussed (e.g., unmeasured hormonal/metabolic factors). Standardized ROI placement and preprocessing were used to reduce technical variability in image analysis. |
| Study size | 10 | Explain how the study size was arrived at |  | No formal power calculation was performed; the final sample reflects all eligible patients during the 10-year period. This is typical of retrospective case–control studies of exploratory nature. |

Continued on next page

| Quantitative variables | 11 | Explain how quantitative variables were handled in the analyses. If applicable, describe which groupings were chosen and why | Methods | FD, lacunarity, MCW, and PMI were treated as continuous variables. Non-parametric tests were used due to non-normal distribution. Variables were not categorized beyond the predefined cholesterol groups. |
| --- | --- | --- | --- | --- |
| Statistical methods | 12 | (*a*) Describe all statistical methods, including those used to control for confounding | Methods | Statistical analysis was performed using IBM SPSS Statistics (version 30.0). Data distribution was assessed using both the Kolmogorov–Smirnov and Shapiro–Wilk tests. As the majority of variables did not follow a normal distribution, non-parametric methods were applied throughout. Group comparisons were conducted using the Kruskal–Wallis test. When this test indicated significant differences, Dunn’s post hoc test with Bonferroni correction was used to identify pairwise differences. Statistical significance was set at p < 0.05. |
|  |  | (*b*) Describe any methods used to examine subgroups and interactions |  | No subgroup or interaction analyses were conducted. The study compared radiographic parameters across three predefined cholesterol-level groups only. Subgroup analysis was not feasible due to sample size and study design. |
|  |  | (*c*) Explain how missing data were addressed |  | No missing data were present in the final statistical dataset. Patients were only included in the analysis if complete laboratory and radiographic data were available, in accordance with the predefined inclusion criteria. |
|  |  | (*d*) *Cohort study*—If applicable, explain how loss to follow-up was addressed  *Case-control study*—If applicable, explain how matching of cases and controls was addressed  *Cross-sectional study*—If applicable, describe analytical methods taking account of sampling strategy |  | Not applicable |
|  |  | (*e*) Describe any sensitivity analyses |  | Not applicable |
| Results | | | | |
| Participants | 13* | (a) Report numbers of individuals at each stage of study—eg numbers potentially eligible, examined for eligibility, confirmed eligible, included in the study, completing follow-up, and analysed | Results | The total sample size was 92 patients, categorized into three cholesterol-level groups. |
|  |  | (b) Give reasons for non-participation at each stage | “Participants” section of the Methods. | Patients were excluded if they had systemic bone-affecting conditions, used medications influencing bone turnover, or had local oral pathology or risk factors that could compromise radiographic assessment or implant outcomes. |
|  |  | (c) Consider use of a flow diagram | Figure 3 | A flow diagram (Figure 3) illustrates the process of patient selection, including inclusion and exclusion steps. |
| Descriptive data | 14* | (a) Give characteristics of study participants (eg demographic, clinical, social) and information on exposures and potential confounders | Table 1 | Descriptive characteristics (age, sex) are provided per group in Table 1, along with total cholesterol categorization. |
|  |  | (b) Indicate number of participants with missing data for each variable of interest | Laboratory assessment | Not all patients had complete laboratory data for all listed parameters. This is stated in the Laboratory Assessment section. However, outcome variables (FD, lacunarity, MCW, PMI) were available for all included patients. |
|  |  | (c) *Cohort study*—Summarise follow-up time (eg, average and total amount) |  | Not applicable |
| Outcome data | 15* | *Cohort study*—Report numbers of outcome events or summary measures over time |  |  |
|  |  | *Case-control study—*Report numbers in each exposure category, or summary measures of exposure | *Results* | *FD, lacunarity, MCW, and PMI values are presented per group and region, with medians, ranges, and significance levels reported in both text and tables (Tables 2–3).* |
|  |  | *Cross-sectional study—*Report numbers of outcome events or summary measures |  |  |
| Main results | 16 | (*a*) Give unadjusted estimates and, if applicable, confounder-adjusted estimates and their precision (eg, 95% confidence interval). Make clear which confounders were adjusted for and why they were included | Results | Group comparisons were unadjusted. Median values and p-values were reported for all outcome variables. No confidence intervals or multivariable models were used, as this was an exploratory, retrospective study. Potential confounding factors (e.g., age, hormonal status) are discussed as limitations, but were not statistically adjusted for. |
|  |  | (*b*) Report category boundaries when continuous variables were categorized | Methods | Total serum cholesterol was categorized into normal (<5.2 mmol/L), borderline high (5.2–6.2 mmol/L), and high (>6.2 mmol/L), following Mayo Clinic and NCEP ATP III guidelines. |
|  |  | (*c*) If relevant, consider translating estimates of relative risk into absolute risk for a meaningful time period |  | Not applicable |

Continued on next page

| Other analyses | 17 | Report other analyses done—eg analyses of subgroups and interactions, and sensitivity analyses |  | No subgroup or multivariable analyses were performed. This is acknowledged in the limitations section of the discussion. |
| --- | --- | --- | --- | --- |
| Discussion | | | | |
| Key results | 18 | Summarise key results with reference to study objectives | Discussion | The discussion begins by restating that only FD in the anterior mandible differed significantly between cholesterol groups, consistent with the study aim. |
| Limitations | 19 | Discuss limitations of the study, taking into account sources of potential bias or imprecision. Discuss both direction and magnitude of any potential bias | Discussion | Limitations include retrospective design, modest sample size, use of total cholesterol only, and lack of stratification for age, sex, or hormonal status. These are thoroughly acknowledged. |
| Interpretation | 20 | Give a cautious overall interpretation of results considering objectives, limitations, multiplicity of analyses, results from similar studies, and other relevant evidence | Discussion | The discussion is balanced and contextualizes findings within existing literature. The possible pathophysiological basis of the FD increase is discussed, with appropriate caution. |
| Generalisability | 21 | Discuss the generalisability (external validity) of the study results | Discussion | The authors acknowledge that results may not generalize to all populations, due to sample size, region specificity, and potential unmeasured confounders. They call for future prospective studies. |
| Other information | |  | | |
| Funding | 22 | Give the source of funding and the role of the funders for the present study and, if applicable, for the original study on which the present article is based |  | This research received no specific grant from any funding agency in the public, commercial, or not-for-profit sectors. |

*Give information separately for cases and controls in case-control studies and, if applicable, for exposed and unexposed groups in cohort and cross-sectional studies.

**Note:** An Explanation and Elaboration article discusses each checklist item and gives methodological background and published examples of transparent reporting. The STROBE checklist is best used in conjunction with this article (freely available on the Web sites of PLoS Medicine at http://www.plosmedicine.org/, Annals of Internal Medicine at http://www.annals.org/, and Epidemiology at http://www.epidem.com/). Information on the STROBE Initiative is available at www.strobe-statement.org.
